# Zoogeography of Intertidal Communities in the West Indian Ocean as Determined by Ocean Circulation Systems: Patterns from the *Tetraclita* Barnacles

**DOI:** 10.1371/journal.pone.0045120

**Published:** 2012-09-14

**Authors:** Ling Ming Tsang, Yair Achituv, Ka Hou Chu, Benny Kwok Kan Chan

**Affiliations:** 1 Simon F. S. Li Marine Science Laboratory, School of Life Sciences, The Chinese University of Hong Kong, Shatin, Hong Kong; 2 The Mina and Everard Goodman Faculty of Life Sciences, Bar-Ilan University, Ramat-Gan, Israel; 3 Biodiversity Research Center, Academia Sinica, Taipei, Taiwan; University of Connecticut, United States of America

## Abstract

The Indian Ocean is the least known ocean in the world with the biogeography of marine species in the West Indian Ocean (WIO) understudied. The hydrography of WIO is characterized by four distinct oceanographic systems and there were few glacial refugia formations in the WIO during the Pleistocene. We used the widely distributed intertidal barnacle *Tetraclita* to test the hypothesis that the distribution and connectivity of intertidal animals in the WIO are determined by the major oceanographic regime but less influenced by historical events such as Pleistocene glaciations. *Tetraclita* were studied from 32 locations in the WIO. The diversity and distribution of *Tetraclita* species in the Indian Ocean were examined based on morphological examination and sequence divergence of two mitochondrial genes (12S rDNA and COI) and one nuclear gene (histone 3, H3). Divergence in DNA sequences revealed the presence of seven evolutionarily significant units (ESUs) of *Tetraclita* in WIO, with most of them recognized as valid species. The distribution of these ESUs is closely tied to the major oceanographic circulation systems. *T. rufotincta* is distributed in the Monsoonal Gyre. *T. ehsani* is present in the Gulf of Oman and NW India. *Tetraclita* sp. nov. is associated with the Hydrochemical Front at 10°S latitude. *T. reni* is confined to southern Madagascan and Mauritian waters, influenced by the West Wind Drift. The endemic *T. achituvi* is restricted to the Red Sea. *Tetraclita serrata* consists of two ESUs (based on mtDNA analysis) along the east to west coast of South Africa. The two ESUs could not be distinguished from morphological analysis and nuclear H3 sequences. Our results support that intertidal species in the West Indian Ocean are associated with each of the major oceanographic circulation systems which determine gene flow. Geographical distribution is, however, less influenced by the geological history of the region.

## Introduction

The West Indian Ocean (WIO) consists of the Indian Ocean, the Arabian Sea and two evaporative basins, the Red Sea and the Persian Gulf, supporting high diversity of marine fauna [1], [2]. In spite of its biological richness, the Indian Ocean remains one of the least known oceanic realms [2]. The complex hydrology and geological history are anticipated to play important roles in determining the present biogeographic pattern in the WIO. The formation of the Indian Ocean occurred more than 180 million years ago (mya) [3]. From 158–160 mya, India, Seychelles and Madagascar were part of the same continent and drifted from East Africa. From 84–96 mya, the India-Seychelles continent was separated from Madagascar and drifted northwards, along the east African coast [4]. About 65 mya, India was separated from Seychelles and collided with Eurasia 55–65 mya (see [4] for details). On the East African coast, Zanzibar Island and Mafia Islands were separated from the shallow waters in the Pleistocene [4]. The Red Sea was formed by two distinct phases of sea floor spreading along the East African rift. The Persian Gulf is a sedimentary basin with very shallow water of about 30 m in depth and is considered a remnant of the Tethys Sea [5]. During the Pleistocene glaciations, when the sea level was lowered, not many refugia were formed in the WIO, compared to the adjacent Western Pacific region [6]. The Persian Gulf was completely dry during the glacial period due to its shallow depth. It was repopulated by Indo-Pacific biota after the connection with the Indian Ocean was reestablished [5]. During the Pleistocene glaciations, the Red Sea was isolated as the shallow connection between the Red Sea and Arabian Sea was constricted by the formation of land bridges, resulting in high endemism in the Red Sea [7].

**Figure 1:**
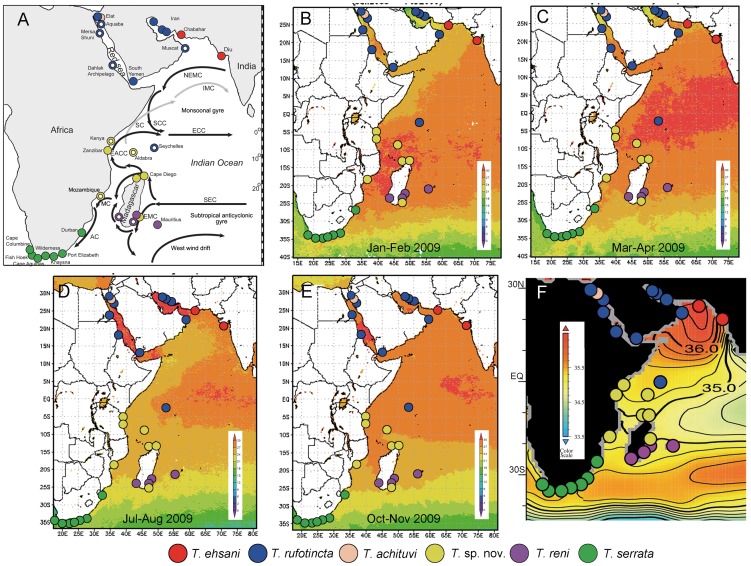
Distribution pattern of *Tetraclita* spp. in relation to physical factors in the West Indian Ocean. A. Sampling localities and biogeography of *Tetraclita* spp. (red – *T. ehsani*, blue – *T. rufotincta*, pink – *T. achituvi*, yellow – *T*. sp. nov., purple – *T. reni*, green – *T. serrata*) in the present study. Closed circles indicate samples with DNA sequences for molecular analysis and open circles indicate museum samples which were used for morphological identifications. NEMC – North East Monsoon Current, IMC – Indian Monsoon Current, ECC – Equatorial Counter Current, EACC – East Africa Coastal Current, SEC – South Equatorial Current, SC – Somali Current, SCC – Somali Counter Current, MC - Mozambique Current, AC –Agulhas Current, EMC – East Madagascar Current. Sea surface temperature map in 2009 of West Indian Ocean produced with the Giovanni online data system (developed and maintained by the NASA GES DISC) showing (B) January-February, (C) March-April, (D) July-August, (E) October-November. (F) Annual average salinity map in 2009 produced with the Marine Ocean Atlas Figures 2009 (NOAA) showing the variation in salinity profiles in the West Indian Ocean.

The complex ocean current pattern in the WIO is also expected to have major influence on the distribution of marine biota. There are a number of unique oceanographic circulation systems [8] prevalent in the WIO, each with distinct hydrochemical parameters including water temperature, salinity, dissolved oxygen and nutrients. In the northern part of the Indian Ocean (north of 10°S), the waters have low dissolved oxygen and high nutrient concentration, as influenced by the Monsoonal Gyre, in which the surface circulation reverses every half year, and the currents inside this system are composed of large eddies (Figure 1A) [9], [10]. Around the latitude of 10°S, there is a strong hydrographical and chemical front (hereafter named as Hydrochemical Front) with lower salinity (35; Figure 1F) and sharp gradient changes in dissolved oxygen, salinity, nitrate and phosphate content, separating the Monsoonal Gyre from the Southern Hemisphere Anticyclonic Gyre. The waters in the Southern Hemisphere Anticyclonic Gyre with high oxygen and low nutrient content are influenced by South Equatorial Current, the Agulhas Current Systems and part of the West Wind Drift (Figure 1A) [8]. The third circulation component in WIO is the Antarctic waters with the Circumpolar Current, which brings cold water of low salinity to the southern part of the Indian Ocean including the South African waters (Figure 1) [8]. The Red Sea and Persian Gulf are relatively isolated basins with high evaporation rate, with inshore seawater of very high salinity (up to 40) and the outflows increase the salinity of the Arabian Sea [8], [11].

In contrast to our relatively comprehensive knowledge on the geological history and ocean current regime, we know little about the biogeography of marine biota in the WIO. Previous biogeographical studies suggest the WIO is divided into northern and southern zoogeographical provinces attributed to great differences in the level of species endemism (see [12] for review; [13]–[16]). Biogeographic analysis on shallow water holothuroids suggests that the WIO can be split into at least three biogeographic units, which partially overlap with the prevalent current systems [16]. The study further provides evidence that contemporary current pattern is of greater importance than past geological events in shaping the observed faunal distribution. However, there is a lack of evidence from other organisms to support this hypothesis and the circumtropical biogeographic pattern of the region remains poorly understood. Moreover, the validity of biogeographic pattern is determined by the accuracy of species identification. Previous diversity studies of species in WIO were based largely on morphological examination that does not reveal the presence of cryptic species. This may lead to underestimation of biodiversity and result in erroneous distribution patterns [17], [18]. Studies on the genetic structure of species in WIO and their relations to the oceanographic patterns and geological history are scarce. Recent molecular studies include those on *Penaeus monodon* [19], periwinkles [20], snappers [21], mangrove crabs [22] and mud crabs [23]. The WIO was not the primary focus of these studies so their sampling effort within WIO is limited and thus could not reflect population differentiation on a fine scale. Therefore, it is essential to have extensive geographical sampling in the Indian Ocean and incorporate both morphological data and molecular markers to further test this hypothesis and evaluate the relative importance of contemporary and historic factors in determining geographical distribution.

Barnacles are good model organisms for coastal zoographical studies because they exhibit high abundance on rocky shores, being the major space occupiers. Their life cycle consists of a sessile adult and a planktonic larval stage. As a result, the dispersal pattern of their larvae and thus the distribution range of the adults, would be expected to reflect their interaction with oceanographic currents [24], [25]. Intertidal barnacles of the genus *Tetraclita* are common and widespread in tropical and subtropical oceans including the WIO [26]. *Tetraclita squamosa rufotincta* was regarded as the only sub-species of *T. squamosa* present in the WIO when Pilsbry identified it in Yemen in 1916, and subsequently recorded it from the northwest coast of India, Red Sea, East African coast and Madagascar [27]–[36]. *Tetraclita serrata* was reported in the South African waters [37], [38]. Ross [39] recognized *T. s. rufotincta* as a distinct species *Tetraclita rufotincta* from *T. squamosa*. Ross [39] identified *T. achituvi* and *T. barnesorum* in addition to *T. rufotincta* from the Red Sea. Further morphological and molecular analysis, however, suggested *T. barnesorum* was synonyms to *T. rufotincta* but *T. achituvi* is a valid species [39]–[41]. In Madagascar, *Tetraclita reni* [42], [43] was distinguished from the hitherto *T. rufotincta* [28], [29] and a few consistent morphological diagnostic characters can be used to distinguish between *T. reni* and *T. rufotincta* [43]. In the Gulf of Oman, *Tetraclita ehsani* was identified from *Tetraclita rufotincta* by having diagnostic differences in tergum morphology [44].

In the present study, we examine the diversity pattern of the intertidal barnacle *Tetraclita* using a combined morphological and molecular approach (sequence divergence of two mitochondrial genes (12S rDNA and COI) and one nuclear gene (histone 3)) from 32 locations in the WIO. Based on the gene flow and distribution pattern obtained, we test the hypothesis whether the biogeography of intertidal barnacles is mainly affected by the major oceanographic patterns and less influenced by geological history in WIO [16].

**Table 1:** Sampling locations, abbreviations and numbers of samples sequenced and morphologically examined for each location.

Locations	Abbreviation	species	mt	H3	Morphology
Elat, Red Sea	EL	*T. rufotincta*	19	8	19
North shores, Elat, Red Sea	EL	*T. achituvi*	7	4	7
Gulf of Aquaba, Red Sea		*T. rufotincta*	nil	nil	10
Mersa Shuni, Egypt		*T. rufotincta*	nil	nil	8
Dahlak Archipelago, Red Sea		*T. rufotincta*	nil	nil	2
Muscat, Oman		*T. rufotincta*	nil	nil	6
Khalf Mukalla, Yemen	YE	*T. rufotincta*	1	1	4
South Yemen		*T. rufotincta*	nil	nil	10
Zanzibar	ZB	*T.* sp. nov.	6	4	8
Bandar Lengeh, Iran	BL	*T. rufotincta*	18	2	18
Bushehr, Iran		*T. rufotincta*	nil	nil	3
Parsian, Iran	PA	*T. rufotincta*	2	2	2
Chabahar, Iran	CH	*T. ehsani*	1	1	2
Diu, India	DI	*T. ehsani*	45	0	50
Cape Diego, Madagascar		*T.* sp. nov.	nil	nil	5
Tanikely, Madagascar	TA	*T.* sp. nov.	3	0	3
Nossy-Kousba, Madagascar		*T.* sp. nov.	nil	nil	2
Fort Dalphin, Madagascar	FD	*T.* sp. nov.	59	19	59
Ambovombe, Madagascar		*T. reni*	nil	nil	1
Connoniers Point, Mauritius	MA	*T. reni*	1	0	5
Kilefe, Kenya		*T*. sp. nov.	nil	nil	2
Passe Gidnnet, Aldabra Atoll		*T.* sp. nov.	nil	nil	10
Mahe Island, Seychelles		*T. rufotincta*	nil	nil	3
Kosi Bay, Mozambique		*T.* sp. nov.	nil	nil	2
Belty Bay, S. Africa	BB	*T. serrata*	7	0	10
Kenton, S. Africa	KE	*T. serrata*	6	1	10
Fish Hoek, S. Africa	FH	*T. serrata*	39	0	42
Durban, S. Africa	DB	*T. serrata*	32	10	9
Knaysna, S. Africa	KN	*T. serrata*	7	0	10
Wilderness, S. Africa	WI	*T. serrata*	8	8	8
Cape Agulhas, S. Africa	CA	*T. serrata*	7	1	11
Cape Columbine, S. Africa	CC	*T. serrata*	8	2	10

Abbreviations designate those locations with molecular data. Site names with barnacles under morphological examination only are not abbreviated.

## Results

### Morphological Diversity of *Tetraclita* Species in the WIO

We have identified six species of *Tetraclita* in the WIO, including one undescribed species (hereafter named *Tetraclita* sp. nov.), based on morphological approach. Each species has diagnostic characters for taxonomic identification. The type locality of *Tetraclita rufotincta* is Yemen and all samples from Yemen, the Iranian coast in the Persian Gulf and Red Sea agree well with the type description by Pilsbry [27]. *Tetraclita ehsani*, a new species recently described [44] collected from Iran and NW India has straight basal margin in the tergum, when compared to the concave basal margin of the tergum in *T. rufotincta*. *Tetraclita* sp. nov. collected from Zanzibar and northern Madagascar differs from *T. rufotincta* by having an elongated antennuliform cirrus III. *Tetraclita reni* from southern Madagascar differs from other species by having a special type of multicuspidate setae in the cirrus III. *Tetraclita achituvi* has diagnostic characters of a narrow base in tergum in combination with antennuliform cirrus III. *Tetraclita serrata* differs from other *Tetraclita* spp. in WIO by having green shells.

### Molecular Analysis

**Figure 2:**
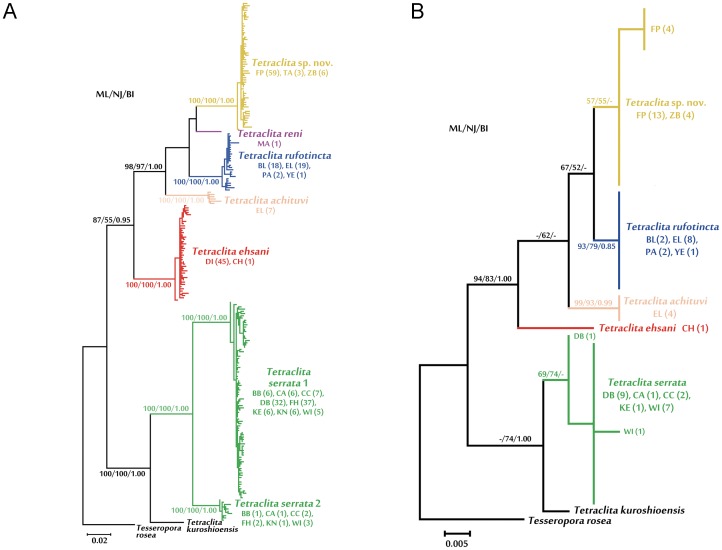
Neighbor-joining tree of *Tetraclita* in the WIO. (A) mtDNA COI and 12S; and (B) nuclear H3 gene sequences. The bootstrap value of NJ and ML, and posterior probability of BI analyses are shown on the corresponding branch for the major nodes for all values >50 in NJ and ML, and >0.75 in BI. The abbreviations of the sampling locations are listed in Table 1. The numbers in the bracket after the location abbreviation indicate the number of sequence collected from that location.

We have determined COI and 12S sequences from a total of 276 individuals of ingroups and also obtained 65 sequences of H3 gene. Sequences were deposited with GenBank (Table 1; Accession nos. JX186199–JX186496). The sequences were aligned and truncated using the same length to minimize the amount of missing data. The aligned COI and 12S datasets are 555 bp and 317 bp in length (872 bp in the combined dataset), respectively. We recovered seven ESUs within the ingroup taxa in the mitochondrial gene tree (Figure 2A). Monophyly of all of the ESUs is strongly supported by all of the three analytical methods (maximum likelihood (ML), neighbor-joining (NJ) and Bayesian inference (BI)).

All samples from the Yemen, Iran and most of the individuals from the Red Sea (Elat) cluster into an ESU, representing *Tetraclita rufotincta* (Figure 2A). The remaining Elat samples belong to *Tetraclita achituvi*. The specimens collected from East Africa (Zanzibar) and Madagascar (both north and south) form another ESU which represents a cryptic species (*Tetraclita* sp. nov.; Figure 2A). *Tetraclita reni* is represented only by a single individual in the molecular analysis, which were collected from Mauritius. The single specimen from Chabahar (Iran) and all samples from Diu (India) are *Tetraclita ehsani* (Figure 2A). These five ESUs group together in the gene tree designated as the *T. rufotincta* group. The corrected net sequence divergences among these clades range from 8.9% to 14.9% in COI and 2.8% to 9.6% in 12S (Tables 2, 3). On the other hand, *Tetraclita serrata*, which consists of two ESUs as revealed by mtDNA analysis, is more closely related to *Tetraclita kuroshioensis*, instead of the other WIO taxa (Figure 2). The two ESUs of *T. serrata* differ by 8.5% and 4.4% in the net sequence divergence of COI and 12S, respectively. The genetic diversity (both haplotype and nucleotide) of the ESUs is high for COI but relatively lower for the more conserved 12S (Tables 5, 6).

**Table 2:** Pairwise genetic divergence among the *Tetraclita* species in the West Indian Ocean and the two outgroups based on COI.

	Tach	Truf	Tren	Tsp	Tehs	Tser1	Tser2	Tkuo	Tros
Tach	0.012								
Truf	0.128	0.009							
Tren	0.111	0.089	n.a.						
Tsp	0.122	0.111	0.095	0.012					
Tehs	0.126	0.133	0.122	0.149	0.009				
Tser1	0.222	0.203	0.207	0.220	0.193	0.010			
Tser2	0.202	0.197	0.199	0.201	0.178	0.085	0.008		
Tkuo	0.206	0.191	0.190	0.184	0.164	0.167	0.149	n.a.	
Tros	0.154	0.138	0.141	0.123	0.164	0.216	0.211	0.194	n.a.

The intraspecific divergence is shown on the diagonal. Tach: *Tetraclita achituvi*; Truf: *Tetraclita rufotincta*; Tren: *Tetraclita reni*; Tsp: *Tetraclita* sp. nov.; Tehs: *Tetraclita ehsani*; Tser1 and 2: *Tetraclita serrata* clade 1 and 2; Tkuo: *Tetraclita kuroshioensis*; and Tros: *Tesseropora rosea*. The intraspecific divergence for Tkuo and Tros is not available as only one specimen was analyzed for each of them.

**Table 3:** Pairwise genetic divergence among the *Tetraclita* species in the West Indian Ocean and the two outgroups based on 12S.

	Tach	Truf	Tren	Tsp	Tehs	Tser1	Tser2	Tkuo	Tros
Tach	0.007								
Truf	0.058	0.004							
Tren	0.041	0.033	n.a.						
Tsp	0.046	0.028	0.028	0.004					
Tehs	0.096	0.083	0.071	0.088	0.002				
Tser1	0.109	0.106	0.091	0.099	0.117	0.004			
Tser2	0.113	0.114	0.094	0.112	0.115	0.044	0.003		
Tkuo	0.094	0.097	0.085	0.096	0.103	0.047	0.046	n.a.	
Tros	0.084	0.070	0.071	0.081	0.085	0.074	0.073	0.065	n.a.

The intraspecific divergence is shown on the diagonal. Tach: *Tetraclita achituvi*; Truf: *Tetraclita rufotincta*; Tren: *Tetraclita reni*; Tsp: *Tetraclita* sp. nov.; Tehs: *Tetraclita ehsani*; Tser1 and 2: *Tetraclita serrata* clade 1 and 2; Tkuo: *Tetraclita kuroshioensis*; and Tros: *Tesseropora rosea*. The intraspecific divergence for Tkuo and Tros is not available as only one specimen was analyzed for each of them.

To further confirm the identity of the putative cryptic species identified in the mitochondrial gene tree, we generated the H3 sequences from a sub-set of samples from each of the seven ESUs identified, except *T. reni* for which the PCR failed for all the samples we had. All except the two *T. serrata* clades revealed in the mtDNA gene tree were found to be monophyletic in the H3 tree (Figure 2B) but the sequence divergence is low (ranging from 0.7% to 2.4% among the four WIO clades; Table 4) as compared to the mitochondrial genes. All but two *T. serrata* examined exhibit identical nuclear H3 gene in spite of high genetic divergence in the mitochondrial genes (Figure 2B). Two specimens from Durban differed by a single substitution from the other individuals. We sequenced five individuals from Wilderness, where the two clades occur with comparable frequency (five clade 1 and three clade 2 out of 8 individuals) plus one individual from Cape Columbine (Figure 2B). The two individuals from Wilderness exhibiting *T. serrata* clade 2 mitochondrial haplotype share identical nuclear H3 sequences with the other individuals from the clade 1 (Figure 2B).

### Biogeography of the *Tetraclita* ESUs in the West Indian Ocean

In order to gain more information on the biogeographical distribution of each ESU, we also used the diagnostic morphological characters of the *Tetraclita* ESUs to identify samples from museum collections which were mostly not suitable for DNA analysis. Combining the results of the molecular and morphological analyses, the distribution of the ESUs could be inferred (Figure 1A, Table 1). *Tetraclita ehsani* is confined to the most eastern part of the WIO, present in the Iranian coast in the Gulf of Oman and Diu in the NW India (Figure 1A). Diu is probably the southern limit of *Tetraclita ehsani* as *Tetraclita* was absent from the coast of Mumbai (BKKC personal observation). *T. rufotincta* is present in the Persian Gulf, along the southern coast of the Arabian Peninsula, covering Oman, Yemen and in the northern and southern Red Sea including the Egyptian coast, Dahlak Archipelago and Gulf of Aqaba. In the Seychelles, based on museum specimens, *T. rufotincta* is present, suggesting it may be the southern limit of *T. rufotincta* (Figure 1A). *T. achituvi*, was only recorded in the Gulf of Aqaba in the northern Red Sea (Figure 1A). *Tetraclita* sp. nov. was recorded from Aldabra archipelago, Kenya and Zanzibar along the east African coast and on the North and SE of Madagascar (Figure 1A). In southern Madagascar (morphological analysis of samples from museum) and Mauritius, *Tetraclita reni* is present. In South Africa, starting from Durban to the SW coast, only *Tetraclita serrata* was observed (Figure 1A). There are two ESUs co-existed in these sites, with one ESU dominated over the other, except at the easternmost site, Durban, which only contains specimens from the dominant ESU (Figure 1A).

### Regional Environmental Differences

#### Water temperature

The waters at the Hydrochemical Front at around 10°S latitude including the Zanzibar, Aldabra and northern Madagascan waters had relatively higher water temperature (28°C) from January to August (Figure 1B–D). From January to August, water temperature in the Monsoonal Gyre covering the Arabian Sea, Gulf of Oman and Gulf of Aden was relatively lower than that around 10°S, which ranged from 24–27°C (Figure 1B–D). In winter (Figure 1E), water temperature in the Monsoonal Gyre and the Hydrochemical Front became similar. The Persian Gulf had greatest annual temperature variation (Figure 1B–D). Water temperature in the Persian Gulf reached above 32°C from July to August, which is the highest value among all WIO sampling sites (Figure 1D), and dropped below 20°C in January and February, which is the lowest temperature among the sampling sites in the northern WIO (Figure 1B–C). In the Red Sea, water temperature in the southern part was consistently higher than the northern part in the annual cycle (Figure 1B–D). In the southern WIO, water temperature decreased gradually along the latitudinal gradient from Anticyclonic Gyre in Madagascan waters to the Circumpolar Current region in South Africa (Figure 1B, D) and such latitudinal gradient in decreasing temperature was similar all year round. Along the Natal to South African coast, Durban had consistently higher water temperature than the Port Elizabeth and the lowest annual temperature in the WIO was recorded in the southwest coast of Africa.

**Table 4:** Pairwise genetic divergence among the *Tetraclita* species in the West Indian Ocean and the two outgroups based on H3.

	Tach	Truf	Tsp	Tehs	Tser	Tkuo	Tros
Tach	0						
Truf	0.014	0					
Tsp	0.014	0.007	0				
Tehs	0.024	0.024	0.017	0			
Tser	0.035	0.035	0.035	0.039	0		
Tkuo	0.039	0.039	0.039	0.035	0.010	n.a.	
Tros	0.046	0.046	0.046	0.042	0.035	0.039	n.a.

The intraspecific divergence is shown on the diagonal. Tach: *Tetraclita achituvi*; Truf: *Tetraclita rufotincta*; Tren: *Tetraclita reni*; Tsp: *Tetraclita* sp. nov.; Tehs: *Tetraclita ehsani*; Tser1 and 2: *Tetraclita serrata* clade 1 and 2; Tkuo: *Tetraclita kuroshioensis*; and Tros: *Tesseropora rosea*. The intraspecific divergence for Tkuo and Tros is not available as only one specimen was analyzed for each of them.

**Table 5:** Genetic diversity of the *Tetraclita* species in the West Indian Ocean based on the mitochondrial COI gene.

Species	N	Na	h	π	*D*	*F*_S_
*T. serrata* 1	104	79	0.99	0.010	−2.08**	−25.29***
*T. serrata* 2	10	10	1.00	0.008	−0.47	−6.42***
*T. achituvi*	7	6	0.95	0.012	−1.00	−0.56
*Tetraclita* sp. nov.	68	51	0.98	0.008	−2.29***	−25.66***
*T. ehsani*	45	37	0.99	0.009	−2.25**	−25.62***
*T. rufotincta*	40	23	0.89	0.009	−1.77*	−10.83***

Number of sequences (N), number of haplotypes (Na), haplotype diversity (h), nucleotide diversity (π), Tajima’s *D*, Fu’s *F*_S_, are shown. p values:

* = p<0.05,

** = p<0.01,

*** = p<0.001.

**Table 6:** Genetic diversity of the *Tetraclita* species in the West Indian Ocean based on the mitochondrial 12S gene.

Species	N	Na	h	π	*D*	*F*_S_
*T. serrata* 1	104	22	0.80	0.004	−1.62*	−19.26***
*T. serrata* 2	10	6	0.78	0.003	−0.82	−3.54***
*T. achituvi*	7	5	0.90	0.006	−0.73	−1.45
*Tetraclita* sp. nov.	68	21	0.65	0.003	−2.46**	−25.81***
*T. ehsani*	45	14	0.71	0.003	−2.24**	−12.02***
*T. rufotincta*	40	15	0.79	0.005	−1.82*	−10.63***

Number of sequences (N), number of haplotypes (Na), haplotype diversity (h), nucleotide diversity (π), Tajima’s *D*, Fu’s *F*_S_, are shown. p values:

* = p<0.05,

** = p<0.01,

*** = p<0.001.

#### Salinity

The Red Sea, Persian Gulf, Gulf of Aden, Gulf of Oman and the Arabian Sea had the highest salinity in the WIO region for the whole year, reaching 36–36.5 all year round (Figure 1F). The salinity at the SW coast of India (from Mumbai to Kochi) had consistently the lowest salinity at about 34 all year round. Salinity at the Equator to the Hydrochemical Front at around 10°S, including the Somali coast, Eastern Africa and northern Madagascar was similar at about 35 all year round. There was a high salinity front south of Madagascar and east of South Africa, with salinity at around 35.5.

## Discussion

### Biogeography of *Tetraclita* in WIO

The spatial structure of *Tetraclita* in the WIO strongly mirrors the major oceanographic systems and environmental conditions in the region. Each of the four major systems, the Monsoonal Gyre, the Hydrochemical Front, the Subtropical Anticyclonic Gyre and the Circumpolar Currents contain different *Tetraclita* species (Figure 1). The species exhibit allopatric distribution, except a narrow overlapping zone between *Tetraclita* sp. nov. and *T. reni* observed in south Madagascar, suggesting that circulation is the principal factor in shaping the biogeography of *Tetraclita* species in the WIO by determining larval dispersal and supply. The 25.0 and 25.8 Sigma-t surface analyses on phosphate content, dissolved oxygen, salinity and chlorophyll *a* concentration also reveal that the four circulation systems exhibit distinct chemical properties and therefore a lack of mixing among these water masses [45]. Accordingly, this would enhance larval retention, resulting in unique species composition within individual systems.

Based on the distribution and composition of shallow water holothurians, the WIO can be split into at least three biogeographical realms, the Red Sea and associated Arab Basin, the asymmetric circumtropical region covering the east African coast to Mozambique, and the southern Africa [16]. Although previous studies in holothurians [16] not resolve the fine pattern within the circumtropical region, subdivisions are evident from taxonomic turnovers. These three putative biogeographic provinces are supported by our present analysis while fine subdivision is further revealed. The congruence of the pattern observed in the two different faunal groups provides strong evidence for the existence and generality of these biogeographic zones, which are mainly caused by oceanographic discontinuities, in the WIO.

Within each ocean current system, the distribution of barnacle species is further determined by the regional environmental factors, including upwelling, water temperature and salinity. For example, *T. rufotincta* and *T. ehsani* are associated with high salinity waters (>35) in Monsoonal Gyre (Figure 1F). *T. rufotincta* dominates the Gulf of Oman yet it is replaced by *T. ehsani* at the entrance of Arabian Gulf, which has long been described as a major biogeographical barrier attributed to the seasonal cold water upwelling along the Arabian Sea coasts of Yemen and Oman [46]–[48].

*Tetraclita* sp. nov. is common in the region around the equator and to the 10°S where the Hydrochemical Front exists. The water temperature around this region is the highest with lower salinity when compared to the northern and southern Indian Ocean (Figure 1B–F) [8]. The southern Madagascan water is located in the region of WIO Anticyclonic Gyre. In this region, *Tetraclita reni* dominates the southern Madagascar and Mauritius, and in Port Dolphin, Madagascar, *Tetraclita* sp. nov. was recorded together with *T. reni*. *T. reni* is absent from northern locations of Madagascar and appears to be a southern oceanic species. The absence of *T. reni* in northern Madagascar is probably related to the pattern of the South Equatorial Current, which flows westwards and hits the east coast of Madagascar and splits into two currents (Figure 1) [49]. The East Madagascar Current flows southwards along the east Madagascar and reaches the southern tip, where it bifurcates again. One branch of the current becomes the North Madagascar Current, flowing northwards along the west Madagascar coast. This current turns anti-clockwise in the Mozambique Channel and forms the Mozambique Current, which eventually fuses with the remnants of North Madagascar Current to form the southward Agulhas Current (Figure 1A) [49]. As a result, there are no currents flowing from south to north Madagascar, accounting for the absence of *Tetraclita reni* in the northern Madagascan waters. *T. reni* is present in south Madagascar and Mauritius, suggesting its distribution is further affected by the West Wind Drift whose waters have higher salinity (Figure 1F).

### Relative Importance of Contemporary Oceanography and Geological History in Shaping WIO Biogeography

Previous studies in Holothurians [16] proposed that the biogeography of the WIO is best explained by the species dispersal capacity and the prevalent current pattern, whilst the role of recent geological history is less important. Our data provide clear support for this hypothesis. The *Tetraclita* species exhibit distinct spatial structure that is highly concordant with the major circulation systems, yet there is in general lack of intraspecific population genetic structuring within their distribution range, with the exception of *T. serrata* in South Africa. This suggests current systems would facilitate larval transport within each system, while reduce gene flow between systems. Furthermore, the species demonstrate high genetic diversity, suggesting a relatively long-standing, stable population. This is consistent with the fact that there are few glacial refugia in the WIO during the Pleistocene and therefore the fauna could persist through the glaciation periods without population fragmentation and local population extinction [50]. The only exception is the Red Sea which was isolated from the WIO during the glaciation periods. This promoted speciation and the Red Sea therefore serves as a glacial refugium with a level of endemism (e.g. *T. achituvi* in the present study [32], [51], [52]), especially in the northern Red Sea. Genetic analyses in the WIO either reveal a panmictic metapopulation [21], [53] or phylogeographic breaks that are consistent with the biogeographic boundary proposed above (e.g. between populations of littorinid *Echinolittorina millegrana* from Madagascar and those from the Red Sea and Oman [20]). Therefore, it appears that the zoogeography of the WIO biota is affected by recent geological events, such as Pleistocene glaciations, to a lesser extent than reported elsewhere. For instance, the Coral Triangle that attracts the most attention in the West Pacific is characterized by large number of archipelagoes and islands. When the sea level was lowered in the Pleistocene glaciation, many land bridges were formed [6], resulting in sharp genetic differentiation in many marine taxa [54], [55].

Postglacial recolonization and gene flow depend on both the biological properties of the biota as well as the prevalent local circulation realms [50], [55], [56]. Accordingly, the mode of larval development, and therefore the potential dispersal ability, is long considered to have a major effect in distribution of marine species. Holothurians with lecithotrophic larvae (non-feeding, shorter larval stage) have more restricted distribution range and the species are more allopatric than those with planktotrophic larvae in the WIO [16]. The larval development of *Tetraclita rufotincta* in the Red Sea have been reported [35], [57], in which the naupliar larvae are lecithotrophic, without the need to feed. Larval development in the naupliar stages takes 6–8 days. Such a development period is shorter than the planktotrophic larval cycle reported for other barnacle species in the Pacific waters, which ranged from 14–21 days [58]–[60]. This suggests long-distance larval dispersal of the *Tetraclita rufotincta* group cannot be achieved due to the limited length of the larval cycle and explains the restricted geographic distributions of *T. rufotincta* group in the WIO. The short-distance dispersal of *Tetraclita* larvae in the WIO is in contrast to the widespread distribution pattern of *Tetraclita squamosa*, *T. kuroshioensis* and *T. japonica* in the west Pacific Ocean [61]. For instance, the *Tetraclita* species found in the Pacific generally take at least 14 days to complete the larval cycle (e.g. *T. japonica* and *T. squamosa* [58]). These Pacific populations have farther larval dispersal and the adult populations in some geographical locations are sympatric with two species on the same shores, but with different vertical zonation patterns [61], [62]. A similar phenomenon is observed in the barnacle *Chthamalus* in the Atlantic Ocean, of which the larvae of the *C. montagui* are smaller in size and with shorter development period than *C. stellatus* [63]. This results in pronounced genetic subdivision between the Mediterranean and East Atlantic populations in *C. montagui*, but not in *C. stellatus* [64], [65]. Hence, the biogeographic zone of the *Tetraclita* barnacles in the Pacific, and regions elsewhere, is generated by both historic and recurrent barriers to gene flow as well as changes in environmental factors (e.g. temperature), while the biota in the WIO is more determined by hydrology and biological characteristics.

### Phylogeography of South Africa

Previous studies on various coastal animals along the South African coast also reveal intraspecific divergent lineage [66]–[68]. The degree of overlapping in the distribution of different lineages is found to be low even in species with planktonic dispersers and highly localized in species with abbreviated larval development or direct development [67]. Oceanography is one major factor postulated to be responsible for the observed divergence [66], [67]. The Atlantic coast of South Africa is influenced by the Benguela Current and the cool upwelling coastal water. The southernmost part of Benguela System is influenced by the warm water of the Agulhas system [14], [69]–[71] as revealed by the temperature gradient from Durban to the Cape Town and to Cape Columbine in the SW African coast (Figure 1B–E). However, the distribution pattern and division of the lineages could not be explained by hydrography alone [68]. Balancing and local selection are shown to contribute to the maintenance of the genetic differentiation as shown by allozyme/microsatellite markers in abalone [66]. The deep divergence observed in the present study and some of the species analysed before suggest that the divergence is ancient [67]. However, the two clades of *Tetraclita serrata* discovered in the present study show widespread, largely overlapping distribution along the South Africa coast, instead of the highly segregated distribution observed in other taxa. From most of the locations, the two clades coexist with clade 1 dominated over the other, except the easternmost Durban, where only the clade 1 was recorded. Furthermore, the shared nuclear H3 genotype suggests a lack of reproductive barriers and high-level introgression in the species after the removal of isolation. This may reflect that the local selection pressure is relatively mild in *Tetraclita serrata* and the species exhibits high level of gene flow along the South African coast despite the circulation pattern. Interestingly, all 20 individuals from Durban belong to clade 1. The absence of the clade 2 from the coast of Natal might be a result of the oceanographic regime of the South African coast. The Natal area is influenced by the rather warm water of the Agulhas Current. On reaching the wide continental shelf of Agulhas Bank, downstream of Port Elizabeth, Agulhas Current changes noticeably [72]. When the Agulhas Current passes the Agulhas Bank, the current overshoots the African continent and, in a convoluted way, protrudes into the South Atlantic Ocean, and there is a thermal drop of the coastal water. It is possible that the less abundant *T. serrata* clade 2 is restricted to the colder waters of the western Cape, or the larvae cannot cross the thermal barrier at the Agulhas Return Current.

### Diversity and Phylogeny of *Tetraclita rufotincta* Group and *T. serrata*

There is increasing effort in characterizing biogeographical regions in the sea for the need of conservation management and ecological study [73]. However, study of the WIO is severely lacking, in spite of its large area. Studies are highly biased regionally and taxonomically. For instance, most investigations are concentrated in South Africa [74], [75]. There are currently constructions and activities related to recreational purposes in the WIO that would affect the diversity of marine species [2]. Therefore, baseline knowledge on the species diversity and distribution is the first step before any action to be taken.

Our present effort represents one of the most comprehensive analyses combining molecular data with morphological characters to clarify the taxonomic identity and geographic range of a major marine taxon in the WIO. Taxonomy of *Tetraclita* barnacles in the WIO was once confusing and underwent numerous revisions with a number of new species described in recent years [43], [44]. We identified five ESUs (including one new species) in the *Tetraclita rufotincta* group and two clades in *Tetraclita serrata* in the WIO, based on the sequence divergence in the mitochondrial COI and 12S genes. As it is not desirable to define species based solely on divergence and monophyly in mtDNA, we test the species status of the ESUs using a nuclear locus, H3 sequence, and confirm the monophyly of most of the mtDNA ESUs. In the *T. rufotincta* group, the five ESUs based on both the mitochondrial and nuclear H3 sequences are divergent and reciprocally monophyletic, with unique phylogeographical pattern and diagnostic morphological characters. These provide strong evidence that the ESUs in the *T. rufotincta* group are distinct biological species. On the South African coast where only *T. serrata* is present, there are two sympatric clades (differences in K2P distance is 8.5% in COI and 4.4% in 12S), but these clades cannot be distinguished by the nuclear H3 marker. From morphological examination, specimens from these two clades are very similar, with no diagnostic morphological characters. This suggests the two clades of *T. serrata* belong to a single species. Our present findings clearly indicate the diversity in the WIO remains largely unexplored, even for the coastal species that are easily encountered like the *Tetraclita* barnacles. The big gap of our knowledge is partially attributed to political reasons that create difficulties in extensive samplings in wide ranges of nations in the region. On the other hand, the relatively limited resource for biodiversity research in most WIO countries also leads to a lack of biological surveys in many areas. Accuracy in taxonomic information and records in literature pose critical intrinsic limit to any previously proposed biogeographic hypotheses concerning the WIO. This may partly explain why previous efforts fail to reveal the biogeographic subdivision on a fine geographic scale [16]. In sum, it is apparent that international collaboration in ecological survey is required in the WIO given the threats from human disturbance [2].

From the phylogenetic analysis, it appears that *Tetraclita reni* and *Tetraclita* sp. nov. are sister taxa, probably related to their close geographical distribution, within the Hydrochemical Front. *T. rufotincta* is sister to the two eastern African and Madagascar species. *T. achituvi* is restricted to the Red Sea and it was probably diverged from the former three taxa through the isolation of the Red Sea during the Pleistocene. *Tetraclita ehsani* from the Arabian Sea is basal in the group, suggesting the colonization of the *Tetraclita rufotincta* group in the WIO might have started from the region around the Gulf of Oman and further diversified to the west. On the other hand, *T. serrata* is more closely related to *T. kuroshioensis* from the western Pacific, in spite of its geographical proximity to the *T. rufotincta* group. This suggests the *Tetraclita* species occurred in the WIO have two independent origins. A comprehensive phylogenetic analysis of *Tetraclita* species in the world’s oceans is needed to address the origin and diversification of this genus.

The existence of five major biogeographic areas in the WIO is evident based on the spatial distribution of *Tetraclita* species and other marine fauna analyzed in previous studies. These provinces are strongly coincident with the major current systems in the region, suggesting a dominant role of oceanography in shaping the WIO biogeography. On the contrary, the geological history exerts less effect on the genetic structure and distribution of biota as compared to other oceans. These might be caused by the biological properties of the animals and the more complex geological history in other marine systems. Furthermore, previous sampling efforts are too fragmentary and/or localized to reveal the biogeographical pattern and the genetic diversity remains poorly explored. These hamper the identification of fine scale biogeographic pattern in the WIO. Generality of the inferred biogeographic zones requires further affirmation from other taxa, but this is no doubt that our results fill in part of the gap in our understanding of marine biogeography and biodiversity in the WIO.

## Materials and Methods

### Sampling Sites

Samples were collected from 19 locations and preserved in 95 or 100% ethanol prior to laboratory analysis (Figure 1, Table 1). The target species is a common intertidal barnacle which is not an endangered or protected species and not protected for all countries collected. All locations are not privately-owned or protected in any way. The only permit required was for the collections in the Red Sea which was provided by the Nature Research Authority, Israel granted to the author Yair Achituv. Collections in Madagascar are from the samples from the Mainbaza Expeditions (Muséum National d’Histoire Naturelle, France). In addition, many specimens are from museum samples in France and UK (see the acknowledgments section in the manuscript for further information.). Additional specimens from other locations in the WIO were obtained from collections in the Natural History Museum, London, UK (NHM), Zoological Museum, Copenhagen, Denmark (ZMUC) and the Muséum National d’Histoire Naturelle, Paris, France (MNHN) (see Table 1). In total, barnacles from 32 geographical locations were analyzed which covered all the major coastlines in the WIO (Figure 1A, Table 1). Samples collected from *in-situ* samplings were used for both morphological and molecular (mitochondrial COI and 12S rDNA, and the nuclear histone 3, H3 gene) analyses to uncover the presence of any cryptic species and to verify the accuracy of morphological identification. Barnacle samples from museum collections were mainly used for morphological examination and provide further information on the distribution range of the species (based on the diagnostic taxonomic characters of the opercular plates and cirral morphology; see [43], [44]).

### Morphological and Genetic Differentiations of Populations among Geographical Locations

The taxonomy of the described *Tetraclita* in the Indian Ocean, including *Tetraclita rufotincta*, *T. achituvi*, *T. reni* and *T. serrata* follows [26], [39], [43] and [44]. We identified an additional undescribed cryptic species from a combined morphology and molecular approach (see Results). The morphology, shape of opercular plates and the arthropodal characters [76], of the cryptic species was investigated and the diagnostic characters for each species were identified.

Total genomic DNA was extracted from whole soft tissue of individual barnacles using the commercial QIAamp Tissue Kit (QIAGEN). Partial sequences of mitochondrial 12S rDNA were amplified using the primer set 12Sai and 12Sbi [77] or FB and R2 [78]. The universal primers LCO1490 and HCO2198 [79] were used to amplify the COI gene. Yet the PCR success rate for the COI of *Tetraclita serrata* samples was low. We subsequently designed a species specific reverse primer, COI-SB 5′ TCAGAATAGATGTTGGTAAAG, which was used with the LCO1490. The amplification of the two genes was conducted in a 25-µl reaction mix containing 5–20 ng of genomic DNA, 1 × PCR reaction buffer, 2 mM MgCl_2_, 200 nM of each primer, 200 µM dNTPs, and 1 unit of *Taq* polymerase (Takara). A PCR profile consisting of 3 min initial denaturation at 94°C, followed by 35 cycles of 30 s at 94°C, 30 s at 48°C, 45 s at 72°C with the final extension for 10 min at 72°C was used for both 12S rDNA and COI. To confirm the species identity of cryptic species, we selected a subset of samples from each of the evolutionary significant units (ESUs) identified by the mitochondrial DNA sequences (see Results) for the nuclear H3 gene sequence analysis, using the primers of [80] with a profile similar to the mitochondrial gene amplification except an annealing temperature of 55°C. The PCR products were then purified using QIAquick gel purification kit according to manufacturer’s instructions (QIAGEN). Sequences were generated using the same sets of primers and determined on an Applied Biosystems (ABI) 3700 automated sequencer using the ABI Big-dye Ready-Reaction mix kit, following the standard cycle sequencing protocol.

Sequences were aligned using MUSCLE [81] with default gap weighting parameters and adjusted by eye. The alignment of COI and H3 sequences was confirmed by translating the aligned DNA sequences into amino acid sequences. The alignment file is available at the website of the journal. The two mitochondrial genes are linked and should share the same evolutionary history [82]. Therefore, we combined the data from the two genes as a single dataset in subsequent phylogenetic analyses. Neighbor-joining (NJ), maximum likelihood (ML) and Bayesian inference (BI) analyses were adopted to the combined mitochondrial gene and the nuclear H3 datasets to determine the phylogenetic relationships between individuals of *Tetraclita* spp. NJ analysis was performed using PAUP* v4.0 b10 [83]. Two other tetraclitid species, *T. kuroshioensis* and *Tesseropora rosea* (collected from Taiwan and Sidney, Australia, respectively), were used as outgroups. Kimura 2-parameter (K2P) distance was used and 1,000 replicates were conducted for bootstrapping (BP). Partitioned analyses were performed for the combined mitochondrial gene dataset to allow differences in the substitution pattern between the two genes in the ML and BI analyses. MrModeltest 2.3 [84] inferred the GTR+I+G was the best-fit model of nucleotide substitution for both the COI and 12S, GTR+I for the H3 gene in the Bayesian analysis. BI analysis was run on MrBayes [85] and two independent runs were carried out with four differentially heated Metropolis coupled Monte Carlo Markov Chains for 5,000,000 generations started from a random tree. Chains were sampled every 500 generations and the first 50% of the trees were discarded as burn-in. A 50% majority-rule consensus tree was constructed from the remaining trees to estimate posterior probabilities (PP). ML analysis was implemented with RAxML 7.0.3 [86]. The model GTRGAMMAI was used for the two partitions (genes) in the mtDNA dataset and the H3 gene dataset, with individual α-shape parameters, GTR-rates and base frequencies estimated and optimized for each partition. We conducted 1,000 BP runs and searched for the best-scoring ML tree. A matrix of the net sequence divergences between ESUs identified (by subtracting intraspecific sequence variability of each of the two ESUs from the mean sequence divergence between the two clades) was also constructed using K2P distance in MEGA v5.1 [87]. Haplotype diversity (h) and nucleotide diversity (π) were estimated for each ESU using ARLEQUIN version 3.0 [88].

### Environmental Data

To assess the relationship between species distribution pattern and environmental factors, oceanographic data were extracted from satellite remote-sensing data and oceanographic atlas to map the species distribution. Oceanographic parameters including surface seawater temperature in Jan-Feb, Mar-April, Jul-Aug and Oct-Nov 2009 were extracted from satellite remote-sensing data in GIOVANNI Database, NASA, USA [89]. Salinity data atlas was obtained from the World Ocean Atlas 2009 as annual averaged salinity at surface water (depth  =  0; WOA 2009, NOAA, see [90]).
